# CORENup: a combination of convolutional and recurrent deep neural networks for nucleosome positioning identification

**DOI:** 10.1186/s12859-020-03627-x

**Published:** 2020-09-16

**Authors:** Domenico Amato, Giosue’ Lo Bosco, Riccardo Rizzo

**Affiliations:** 1 Dipartimento di Matematica e Informatica, Università degli studi di Palermo, Via Archirafi, 34, Palermo, 90123 Italy; 2 Dipartimento di Scienze per l’Innovazione tecnologica, Istituto Euro-Mediterraneo di Scienza e Tecnologia, Via Michele Miraglia, 20, Palermo, 9039 Italy; 3 CNR-ICAR, National Research Council of Italy, Via Ugo La Malfa, 153, Palermo, 90146 Italy

**Keywords:** Nucleosome classification, Epigenetic, Deep learning networks, Recurrent neural networks

## Abstract

**Background:**

Nucleosomes wrap the DNA into the nucleus of the Eukaryote cell and regulate its transcription phase. Several studies indicate that nucleosomes are determined by the combined effects of several factors, including DNA sequence organization. Interestingly, the identification of nucleosomes on a genomic scale has been successfully performed by computational methods using DNA sequence as input data.

**Results:**

In this work, we propose CORENup, a deep learning model for nucleosome identification. CORENup processes a DNA sequence as input using one-hot representation and combines in a parallel fashion a fully convolutional neural network and a recurrent layer. These two parallel levels are devoted to catching both *non periodic* and *periodic* DNA string features. A dense layer is devoted to their combination to give a final classification.

**Conclusions:**

Results computed on public data sets of different organisms show that CORENup is a state of the art methodology for nucleosome positioning identification based on a Deep Neural Network architecture. The comparisons have been carried out using two groups of datasets, currently adopted by the best performing methods, and CORENup has shown top performance both in terms of classification metrics and elapsed computation time.

## Background

The eukaryote genome is packed as chromatin [1], the fundamental unit of packaging is called nucleosome, and it consists of a histone octamer where about 147 bp of DNA is wrapped. Nucleosomes are separated from each other by sequences of DNA called linker DNA. Starting from this low-level organization, chromatin is coiled into many higher-order structures to finally form the chromosomes. Nucleosome positioning indicates the physical packaging of DNA driving the determination of the final architecture of chromatin in the cell [2, 3] both trough the DNA sequence itself and the interaction of other factors, including remodelling proteins [4–6], histone acetylation [7] and others [8]. The chromatin architecture of eukaryotic gene promoters is generally characterized by a nucleosome-free region, where nucleosomes frequently occupy specific positions. For this reason, nucleosomes affect gene regulation shaping the accessibility of transcription factors to occupy their binding sites [9, 10].

Furthermore, nucleosomes influence also the accessibility of different regulative element to DNA, that are critical for other biological processes such as replication [11] and recombination [12]. For these reasons, understanding the structure and function of nucleosomes is of great interest in biology.

The role of DNA sequence in causing nucleosome positions is clear from in vitro studies. Different DNA sequences show different affinities for the histone core. Early studies showed that many nucleosomal related sequences contain quasiperiodic nucleotide distributions [13–15] A comparison of nucleosome sequence maps in vivo and reconstituted in vitro exposes that the relative occupancies of each position are not the same [16]. This in part indicates that nucleosomes in vitro are not regularly spaced, unlike nucleosomes in vivo. These observations led to the conclusion that genomic DNA may encode nucleosome positions [17] opening the possibility to study combinatorial properties of DNA string related to nucleosome preference [18–20]. Recent studies have posed a limit to this deduction, the DNA sequence is for sure an important determinant in nucleosome positioning, but additional factors are needed to determine long-range chromatin organization [21]. The recent MNae-seq experimental approach in nucleosome mapping has provided to the communities several high-resolution nucleosome maps. In 2019 these data have been systematically collected into a database, named NucMap [22]. NucMap is an online database which includes 798 experimental data from 477 samples across 15 species, also supplying a set of very useful tools to visualize and compare the data. These high-resolution data leads to the development of many computational methodologies able to successfully process sequence information to predict the nucleosome presence[17, 23–26]. Taking into consideration these biological studies, and differently from other studies proposed so far, this work intends to try to understand at which extent the DNA sequence is solely responsible for nucleosome positioning. This investigation is carried out by a machine learning model, in particular a deep neural network, which processes only sequence information. In the past years, we gave several contributions to the study of deep learning networks for sequence classification [26–30]. In this work we present *CORENup*, an extension of our previous models by the integration of two different neural networks, each specialized in extracting specific features from sequences, i.e. *non periodic* and *periodic* features. For sure the automatic identification of nucleosome positions seems to have attracted several machine learning researchers, and very effective models have been proposed so far [23, 25, 31, 32]. Among the most performing ones, we have to mention iNuc-PseKNC [23]. It uses a Support Vector Machine with a radial basis function kernel and a novel feature-vector that incorporates six DNA local structural properties. Cross-validation tests on the three benchmark datasets have shown accuracy rates greater than 79%. The work posed a baseline for the machine learning methods, also providing three benchmark dataset very useful for the comparisons. The main issue of the method is that it needs a feature extraction phase for the sequence processing, i.e. the representation for the input data involve a specific preprocessing stage. Conversely, other authors have used the simple *one hot representation* obtaining greater accuracy, using deep neural network classifiers [25, 26, 28]. Actually the model called *Le-Nup* [25] is the top performer. In this work, we will compare CORENup and LeNup in terms of performance metrics, the complexity of the models, and elapsed computation times.

## Methods

Machine learning systems need a mathematical representation of the input objects. This representation is usually obtained defining some quantitative features of the objects and reporting the corresponding measurement results in a representing vector. The choices made during the development of these representations can affect the performance of the whole system. Deep learning techniques can develop a representation of the input data without human guidance, this is important in many classification problems, for example in image processing where it is difficult to describe which features are suitable for a precise task. This property is useful in sequence classification, where it is very hard to spot useful features, and this is one of the reasons why deep learning networks are used.

In the following sections, after the introduction of the sequence representation, we report the motivation of the CORENup architecture, compared with another deep learning neural network used for sequence classification.

### Sequence representation

Machine learning algorithms process *tensor data*, so that DNA sequences should be converted to numerical representations. Fasta files containing DNA sequences are constituted by a set of strings from a finite alphabet *Σ*. This alphabet is restricted to four symbols, e.g. *Σ*={*A*,*C*,*G*,*T*}, corresponding to the 4 bases adenine, cytosine, guanine, thymine, if there is no uncertainty on base value for a specified sequence position, otherwise two or more alternative base values for a single position can be represented using the IUPAC notation, where, for example, the symbol W in a position stands for A or T.

**Fig. 1:**
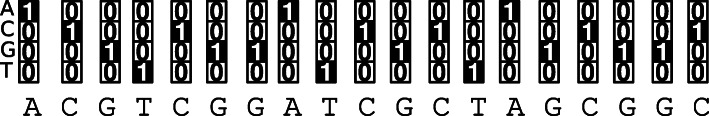
The one-hot representation A simple visualization of the build process for an one-hot representation

In this work the used sequences are from a 4-letters alphabet and sequence representation is the *one-hot encoding*, that transforms a sequence of length *L* into a matrix of dimension (4,*L*). A sketch of the one-hot representation is in Fig. 1. Matrix rows correspond to symbols in the alphabet, while columns indicate the positions in the sequence where the symbols are present. The matrix is binary, i.e. each column *j* have all zero values, but one in the row of the corresponding symbol.

The one-hot representation is a sparse binary representation, suitable only for datasets made of fixed-length small sequences. The main advantage of this sequence representation is that the context of each position, i.e. the sequence of symbols, is preserved and this property will be exploited in the following.

### CORENup neural network model

In nucleosome-linker classification one of the most recent and effective networks is the LeNup network [25]. This network, as many deep learning systems, has a structure inspired by the Google Inception network [33]. These systems are based on a cascade of cells made by convolutional layers in parallel. These convolutional layers have many kernels of dimension 3 or 5, that process the signal in parallel. The basic idea is that in sequence recognition problems it is necessary to look at sequence features obtained at different scales, and then integrate these features so that the next stage can extract more abstract information. LeNup and Inception networks use this approach to obtain a multi-level multi-scale representation of the input, and after many processing cells, these networks use this representation as input for the fully connected final layers. In these networks the number of parameters (weights of the neural network) can grow very quickly, in fact, the LeNup network has more than two millions of parameters.

The integration of many features from layer to layer is the basic idea that also inspired this work, but we wanted to combine different sets of features coming from many sources. In the past, we investigated the use of convolutional and recurrent neural networks for sequence classification [27, 28]. We found that the two methods build two different sets of features from the sequence and that these features can be integrated to build a better classifier.

In the following subsections, the mechanism of convolutional and recurrent (LSTM) networks are introduced, and the combination of the two in the CORENup network is explained.

#### Convolutional neural networks

In a convolutional layer each neuron has a receptive field that scans the input and during this scan builds the layer output. Assuming that the layer has *N* neurons, the receptive field of neuron *i* with $i= 1, 2, \dots N$ has a set of weights $w^{i}_{u}$, with *u*=−*n*,⋯,*n*, where 2*n*+1 is the width of the receptive field of the neuron, and scans an input vector **x**∈ℜ^*d*^; given that the non-linearity of the neuron is a generic function *ϕ*, the output of the neuron $y^{i}_{k}$, associated to the component *k* of the input vector **x**∈ℜ^*d*^ is:

1 $$ y^{i}_{k} = \phi \left(\sum_{u=-n}^{n} w^{i}_{u} * x_{k-u} \right) \\ i= 1,2,\cdots N \\ k = n, n+1, \cdots, d-n  $$

**Fig. 2:**
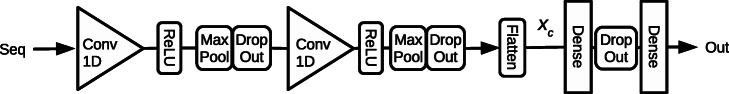
The ConvNet network. The convolutional network used for the classification in [27]

In the architecture in Fig. 2, the first convolutional layer extracts from the sequences features that are contained in a narrow window, the combination of these features is processed by the second convolutional layer. The convolutional layer is stateless and the output depends only on the present input values.

The non-linear function *ϕ* is usually the Rectified Linear Unit (ReLU), defined as:

2 $$ ReLU(x) = \left\{\begin{array}{ll} x \ if \ x \ge 0 \\ 0 \ if \ x < 0 \end{array} \right.  $$

In the work [27] we used these network to classify genetic sequences with good results, and this justifies its use in the CORENup architecture.

#### LSTM network

Recurrent neural networks have a state value, a sliding window scans the input vector and each input vector component updates an internal network state that contributes to generating the output signal. Long Short-Term Memory layer (LSTM) is a particular kind of recurrent neural network where special units called *gates* select the relevant input values used to update the network hidden state. In general LSTM layers process sequences exploiting information that is into the whole sequence, or into a very large window. While the convolutional layer process the whole input pattern in a single step, LSTM process the input pattern exploiting the sequence of features so that we have to introduce the time in the notation and we refer to the input *x* as the input at the time *t*, *x*_*t*_. Assuming *x*_*t*_∈ℜ^*d*^ the LSTM has three gate quantities to take into account: the *forget* gate *f*_*t*_∈ℜ^*u*^, the *update activation* gate *i*_*t*_∈ℜ^*u*^, the *output activation* gate *o*_*t*_∈ℜ^*u*^, where *u* is the number of hidden units of the network. These gates are all functions of the input *x*_*t*_ and of the hidden state of the network *h*_*t*−1_∈ℜ^*u*^; the *h*_*t*_ vector is also the output of the network. These activation are obtained from the following equations:

$$\mathbf{f_{t}} = \sigma\left(\mathbf{W^{f}}\mathbf{x_{t}} + \mathbf{U^{f}}\mathbf{h_{t-1}} + \mathbf{b^{f}}\right) $$

$$\mathbf{i_{t}} = \sigma(\mathbf{W^{i}}\mathbf{x_{t}} + \mathbf{U^{i}}\mathbf{h_{t-1}} + \mathbf{b^{i}}) $$

$$\mathbf{o_{t}} = \sigma(\mathbf{W^{o}}\mathbf{x_{t}} + \mathbf{U^{o}}\mathbf{h_{t-1}} + \mathbf{b^{o}}) $$

where *W*^*f*^,*W*^*i*^,*W*^*o*^ are all weights matrices of dimension (*u*,*d*), and *U*^*f*^,*U*^*i*^,*U*^*o*^ are weights matrices of dimension (*u*,*u*). The gate values are obtained from the current input *x*_*t*_ and the past hidden state value *h*_*t*−1_. The current value of hidden state *h*_*t*_, is obtained from the cell state signal *s*_*t*_ that collects the values of the forget gate, the update gate, and the output gate, and is updated by using:

$$\mathbf{s_{t}} = \mathbf{f_{t}} \odot \mathbf{s_{t-1}} + \mathbf{i_{t}} \odot \tanh(\mathbf{W^{c}}\mathbf{x_{t}} + \mathbf{U^{c}}\mathbf{h_{t-1}} + \mathbf{b^{c}}) $$

**Fig. 3:**
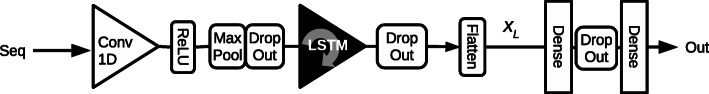
The LSTM network. The architecture with an LSTM network used in [26]

The output of the network is calculated by using the following equation:

$$\mathbf{h_{t}} = \tanh(\mathbf{s_{t}}) \odot \mathbf{o_{t}}\\ $$

where the symbol ⊙ indicates the multiplication element by element. In a preceding work [26] we found that substituting a convolutional layer with an LSTM layer can improve the performance in sequence classification tasks, and the resulting network is in Fig. 3. In this case, the LSTM works on sequence features obtained by the convolutional layer, and this can give better results.

#### Merging the architectures: the CORENup network

**Fig. 4:**
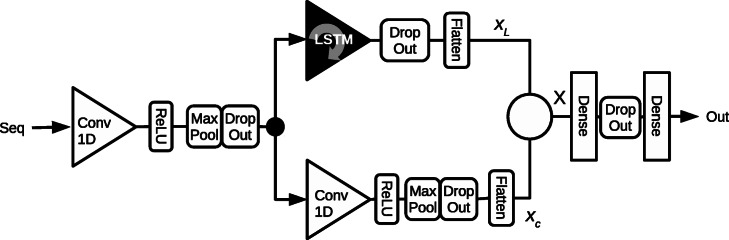
The CORENup architecture. A representation of the CORENup architecture presented in this paper. The details of the architecture are reported in Table 1

As said before both the architectures have advantages so that we decided to integrate the two approaches in the network architecture in Fig. 4. The CORENup network is composed by an input convolutional layer, that extracts some raw features from the sequence, followed by two processing paths: convolutional and recurrent. The combination of these two processing paths is obtained putting side to side the output vectors from each path. Both paths output a 3D tensor, after the flatten layers the two-column tensors *x*_*L*_ and *x*_*c*_ are 1D tensors with different dimensions:

3 $$ \begin{aligned} \mathbf{x_{L}} \in \Re^{L} \\ \mathbf{x_{c}} \in \Re^{c} \end{aligned}  $$

**Table 1:** CORENup structure

**Features extraction path**					
Layer	Kernel Dim	# Hidden Units	stride Dim	Output Dim	# Params
Conv1D	5	50	1	147x50	1.050
MaxPool1D	-	-	2	73x50	0
Dropout 50%	-	-	-	73x50	0
**LSTM Path**					
Layer	Kernel Dim	# Hidden Units	stride Dim	Output Dim	# Params
LSTM	-	50	-	73x50	20.200
Dropout 50%	-	-	-	73x50	0
Flatten	-	-	-	3.650x1	0
**Convolutional path**					
Layer	Kernel Dim	# Hidden Units	stride Dim	Output Dim	# Params
Conv1D	10	50	1	73x50	25.050
MaxPool1D	-	-	2	36x50	0
Dropout 50%	-	-	-	36x50	0
Flatten	-	-	-	1.800x1	0
**Dense path**					
Concatenate	-	-	-	5.450x1	0
Dense	-	370	-	370x1	2.016.870
Dropout 50%	-	-	-	370x1	0
Dense	-	1	-	1x1	371
**Parameters count**					
			**# Parameters**		
Features Extraction Path			1.050		
LSTM Path			20.200		
Convolutional Path			25.050		
Dense Path			2.017.241		
**Total**			2.063.541		

Notice that the dense layer contains the majority of the network parameters

The two vectors are combined to obtain the tensor **X** :

4 $$ \mathbf{X}= \left[ \begin{aligned} \mathbf{x_{L}}\\\mathbf{x_{c}} \end{aligned} \right]  $$

where **X**∈ℜ^*L*+*c*^ is a column vector.

The proposed CORENup network is not so deep compared to the LeNup network: the input signal moves across 4 layers, considering the parallel as two layers and including the fully connected ones, while LeNup has 5 gated convolution layers plus two fully connected. The CORENup spreads more in width than in-depth, but, due to its structure, it can obtain similar or better performance with smaller training time. The details of the CORENup structure are reported in Table 1, where it is also reported the number of the parameter of the network.

The CORENup has roughly the same number of parameters of the LeNup, but the architecture is quite different. The number of layers is less and the majority of the weights is concentrated on the first dense layer because the number of dimension of **X** is *L*+*c* where *L*=3650 and *c*=1800. Considering 370 hidden units in the first hidden layer, we have more than 2 millions of weights concentrated in just one layer. In the LeNup network, as well as other deep neural networks, the weights are spread over many layers so that updates require many calculations, due to the more backpropagation steps, while in the CORENup network the weights in the fully connected layer will be updated in a single back-propagation step.

### Datasets and training details

To be comparable with state of the art methods, the CORENup network was tested using two group of datasets and two different folding techniques. Each of the two groups of datasets has a reference paper where the data have been collected and used to train one or more machine learning models. Adopting such datasets give us the possibility to compare CORENup with other methods, assuming to use the same experimental protocol of the other methods.

The first group of datasets is composed of four sets of DNA sequences. The first three underly nucleosomes from Homo Sapiens (HS), Caenorhabditis Elegans (E) and Drosophila Melanogaster(DM). The details about how these data have been collected can be found in the paper by Guo et al. [23] and in the references therein. The fourth dataset is about Saccharomyces Cerevisiae (Y) and is introduced in [34].

The best performer on this data is the LeNup neural network [25]. Authors provide the source code of the method (githubrepository ), so that we have decided to run the experiments also using this methodology.

Following the experimental protocol reported in the LeNup work [25] we used a 20-fold cross-validation method for each dataset of the first group. The cardinality of each class, for each dataset, is reported in Table 2. For each iteration, we use 1 fold to test model and the remaining 19 folds to train both CORENup and the state of art LeNup network.

**Table 2:** Number of samples in the first group for each class

	Nucleosome	Linker	Total
HS	2273	2300	4573
DM	2900	2850	5750
E	2567	2608	5175
Y	1880	1740	3620

HS represents the Homo Sapiens group; DM represents Drosophila Melanogaster group; E represents the Elegans group; Y represents Yeast group

The second group of datasets give us the possibility to test the prediction methods on different sequence classes of HS, DM and Y species. Such classes include whole-genome (WG) and promoter (PM) sequences of Y, and the largest chromosome (LC), promoter (PM) and 5’UTR exon regions (5U) sequences from DM and HS. The dataset is downloadable in terms of bed file as reported in the reference paper [35]. To collect the sequences, we have used the coordinates in the bed files to fetch the nucleosomal and linker sequences using the genome files downloaded from the UCSC Table Browser. The distribution of the elements in the classes for this group of datasets is shown in Table 3. These datasets were used as a benchmark for different methods available in literature [35]. The protocol used by the author in the work, consisted in the extraction with replacement, for each dataset, of 100 samples of 100 sequences each. We decided to adopt the same protocol for CORENup and LeNup network. Every dataset was split in training and test sets a priori, in such a way that we had sufficient data to train a strong model and a large enough pool from which to extract 100 test samples of 100 items each.

**Table 3:** Number of samples in the second group for each class

	Nucleosome	Linker	Total
HS - LC	97209	65563	162772
HS - PM	56404	44639	101043
HS - 5U	11769	4880	16649
DM - LC	46054	30458	76512
DM - PM	48251	28763	77014
DM - 5U	4669	2704	7373
Y - WG	39661	4824	44485
Y - PM	1880	4463	31836

The labels HS, DM and Y have the same meaning as in Table 2. LC represents the longest chromosome; P represents the promoter sequences; 5U represents the 5UTR Exon region; WG represents the whole genome

CORENup has been implemented using Tensorflow with Keras Backend API [36] environment. It has been trained using the *Adam* optimizer [37] for the computation of the stochastic gradient descent with binary cross-entropy as the loss function. Learning Rate has been set to 3×10^−4^ and, to prevent overfitting, and L2 Regularisation with *λ*=1×10^−3^ has been used. CORENup set the maximum number of epochs to 200 and used an Early Stopping rule for training that stops if the loss function, calculated on validation, doesn’t decrease for 5 consecutive epochs. The size of the validation has bee set to 10% of the training set. The output of both models ranged between 0 and 1, has been used to predict labels with a threshold of 0.5. Every output above this last one was classified as a nucleosome otherwise as a linker.

## Results

Considering the same processing principle of feature integration shared by CORENup and LeNup, and the similar number of parameters, we decided to compare our model with the LeNup network using the two groups of datasets introduced before. The results obtained from the first dataset are evaluated by using the typical metrics of the classification evaluation. For each of the 20-fold the accuracy (ACC), sensitivity (SENS), specificity (SPEC), Matthew’s correlation coefficient (MCC) are evaluated using the following formulas:

$ACC = \frac {TP + TN}{TP + FP + TN + FN}$

$SENS = \frac {TP}{TP + FN}$

$SPEC = \frac {TN}{TN + FN}$

$MCC = \frac {TP * TN - FP * FN}{\sqrt {(TN + FN)*(TN + FP)*(TP + FN)*(TP + FP)}}$where TP is true-positive, FP is false positive, TN is true negative and FN is false-negative. We indicate as positive the nucleosome class. We also calculated the Roc Curve to compare the prediction performance of the four methods and we reported the Area Under the Curve (AUC). The first set of results is related to the HS, DM, E and Y datasets, and in Table 4 the results obtained from two models used in our recent works, indicated as simple LSTM [26] and ConvNet [27] are reported, together with the CORENup and the LeNup networks. The two simpler networks have almost always worst performance than the more complex networks, confirming that with more parameters it is possible to improve the performance of the classifier. Looking at the CORENup column it is possible to see that the CORENup network outperforms the LeNup almost in all the cases; there are just two values below the LeNup performance in Human (HS) and Elegans (E) datasets.

**Table 4:** Experimental results for the 20-Fold procedure with the first group of data-sets

		LSTM	ConvNet	CORENup	LeNup
HS	ACC	0,836 ±0,03	0,83 ±0,03	**0,881** ±0,04	0,873 ±0,02
	SENS	0,898 ±0,03	0,867 ±0,03	**0,931** ±0,06	0,839 ±0,03
	SPEC	0,792 ±0,03	0,814 ±0,03	0,843 ±0,02	**0,906** ±0,04
	MCC	0,681 ±0,002	0,666 ±0,0	0,758 ±0,07	**0,762** ±0,03
	AUC	0,92 ±0,03	0,91 ±0,03	**0,93** ±0,03	0,928 ±0,01
DM	ACC	0,854 ±0,04	0,838 ±0,04	**0,882** ±0,02	0,875±0,02
	SENS	0,872 ±0,03	0,816 ±0,03	**0,898** ±0,02	0,876±0,03
	SPEC	0,841 ±0,05	0,838 ±0,06	**0,869** ±0,02	0,74 ±0,13
	MCC	0,71 ±0,003	0,68 ±0,003	**0,766** ±0,04	**0,766** ±0,04
	AUC	0,93 ±0,02	0,92 ±0,02	**0,94** ±0,02	0,937 ±0,02
E	ACC	0,897 ±0,03	0,895 ±0,03	**0,915** ±0,05	0,912±0,02
	SENS	0,938 ±0,03	0,924 ±0,02	**0,958** ±0,03	0,885±0,02
	SPEC	0,865 ±0,04	0,874 ±0,04	0,882 ±0,03	**0,939** ±0,02
	MCC	0,799 ±0,002	0,795 ±0,001	**0,835** ±0,07	0,832±0,03
	AUC	0,96 ±0,02	0,96 ±0,02	**0,96** ±0,02	**0,96** ±0,02
Y	ACC	0,996 ±0,05	0,996 ±0,06	**1,0** ±0,002	1,0 ±0,0
	SENS	0,998 ±0,05	0,998 ±0,05	0,999 ±0,005	**1,0** ±0,0
	SPEC	0,995 ±0,07	0,995 ±0,08	**1,0** ±0,0	1,0 ±0,0
	MCC	0,992 ±0,003	0,993 ±0,002	0,999 ±0,005	**1,0** ±0,0
	AUC	0,99 ±0,0	0,99 ±0,0	0,99 ±0,0	**1,0** ±0,0

The two networks LeNup and CORENup outperform the simpler networks in Figs. 2 and 3. Best values are shown in boldface

## Discussions

The obtained results sustain the idea that the architecture is important and simply adding more weights, for example making the network deeper, do not automatically improve the performance. In CORENup network the majority of the parameters are used on the first fully connected layer, where it is necessary to integrate features of different nature, non-periodic features obtained from the stateless convolutional layer, and periodic features from the recurrent LSTM layer, and even if they are more than 2 million they have a small impact on the training time. The first 4 rows of Tables 5 and 6 report the training time for the two networks on datasets HS, DM, E and Y. From Table 5 it is possible to see that the CORENup is about three times faster than the LeNup, while Table 6 reports the total training time until the algorithm stops. In two cases this time is longer for the LeNup, because the CORENup keeps improving its performance for more epochs, and the algorithm does not stop the training if there are still improvements on the classification results.

**Table 5:** Time for Epochs

	CORENup	LeNup	Overhead
HS	5	16	0.31
DM	6	20	0.30
E	5	18	0.27
Y	4	12	0.33
HS - LC	63	233	0,27
HS - PM	44	158	0,28
HS - 5U	10	35	0,29
DM - LC	47	171	0,27
DM - PM	57	211	0,27
DM - 5U	4	13	0,30
Y - WG	36	124	0.29
Y - PM	24	88	0.27

Comparison between CORENup and LeNup time for epochs, all the time are expressed in seconds. The Overhead column reports the ratio between CORENup and LeNup times

**Table 6:** Training time

	CORENup	LeNup	Overhead
HS	166,79	305	0,55
DM	219,14	382,75	0,72
E	207,40	312,35	0,66
Y	287,46	186,5	1,54
HS - LC	2544,14	1426	1,78
HS - PM	1558,92	1806	0,86
HS - 5U	316,08	750	0,42
DM - LC	2025.52	4435	0.46
DM - PM	1823.39	3040	0.60
DM - 5U	121.78	171	0.71
Y - WG	791.37	1294	0.61
Y - PM	500.37	1311	0.38

Comparison between CORENup and LeNup Training time, all the time are expressed in seconds. The Overhead column reports the ratio between CORENup and LeNup times

The other eight datasets (HS-LC, HS-PM, HS-5U, DM-LC, DM-PM, DM-5U, Y-WG and Y-PM) are much more difficult because there is much more noise in the sequences. In this trial, the CORENup gives better results on 5 datasets out of 8, as reported in Table 7, still maintaining the same, very fast, training time. Notice that the AUC in dataset HS-PM is only 1∗10^−3^ below the LeNup value and that the only significant differences are on the HS-LC and Y-PM datasets. The training times are reported in the last eight rows of Tables 5 and 6, again it is possible to see that the time for epoch is one-third of the LeNup training time and that the whole training time is still lower, except for the HS-LC dataset.

**Table 7:** Experiments result for the second group of data-sets

	CORENup	LeNup	Best for [35]
HS - LC	0,912	**0,926**	0,65
HS - PM	0,875	**0,876**	0,67
HS - 5U	**0,758**	0,732	∼0,7
DM - LC	**0,734**	0,724	∼0,7
DM - PM	**0,738**	0,734	∼0,7
DM - 5U	**0,746**	0,695	∼0,7
Y - WG	**0,968**	0,939	0,77
Y - PM	0,909	**0,933**	0,79

The AUC values are calculated as explained in the work [35] where the data-sets were originally proposed. The last column reports the results of the best performer among the 8 methods compared in the original paper. Best values are shown in boldface

The third column in Table 7 shows the AUC values of the best performer among the 8 methods compared in the paper by Liu *et al* [35]. The symbol ’ ∼’ is used to indicate a ’very close to’ value, and this approximation is mandatory since the paper reports no more than a bar plot for the AUC values. The comparison shows the improvements obtained by using the two deep neural networks.

All the experiments reported ran on Intel i7 CPU with 32GB of memory RAM and an NVIDIA TITAN V with 12GB of GPU dedicated memory.

## Conclusions

Deep neural networks are suitable for sequence classification because they can automatically extract the useful features from sequence and can use them for classification. In deep neural networks like GoogLeNet [33] or LeNup network [25], these features are processed by a sequence of layers that groups them and extract from this composition new high-order more complex features.

The same principle of feature composition can be exploited using features of different nature, such as the one extracted by convolutional networks and recurrent networks. The convolutional layers can extract and process features that are related to the presence of “static” patterns in the sequences, such as few letters words or other patterns. The recurrent layer can complete the extraction of the feature related to the periodic characteristics of the sequences probably related to word repetition. Using this approach, the input signal follows different processing paths aimed to extract different information from the input. The resulting network is more "wide" than deep, with many layers that work in parallel on the same input. In this paper, we have proposed a deep neural network, called CORENup, which follows this parallel layer composition. CORENup has shown to be a top performer with a smaller training time with respect to the state of the art. This kind of architecture shows that the features extraction and composition process can be obtained not only in deep stacks of convolutional layers but also in shallow modules that process the signal in different ways and the collecting the results in a single representing vector.

Future efforts will be focused to increase the information extracted from the input and to mix them to obtain a more rich input signal for the fully connected layers.

## Data Availability

The source code of CORENup is freely available at https://github.com/DeepLearningForSequence/ CORENup-A-Combination-of-Convolutional-and-Recurrent-Deep-Neural-Networks-for-NucleosomePositioning. The data used for the experiments are downloadable at https://github.com/DeepLearningForSequence/CORENup-Datasets/tree/master/Datasets.

## References

[1] Ridgway P, Almouzni G (2001). Chromatin assembly and organization. J Cell Sci.

[2] Weiner A, Hughes A, Yassour M, Rando OJ, Friedman N (2010). High-resolution nucleosome mapping reveals transcription-dependent promoter packaging. Genome Res.

[3] Whitehouse I, Tsukiyama T (2006). Antagonistic forces that position nucleosomes in vivo. Nat Struct Mol Biol.

[4] Cairns BR (2005). Chromatin remodeling complexes: strength in diversity, precision through specialization. Curr Opin Genet Dev.

[5] Sala A, Toto M, Pinello L, Gabriele A, Di Benedetto V, Ingrassia AMR, Lo Bosco G, Di Gesù V, Giancarlo R, Corona DFV (2011). Genome-wide characterization of chromatin binding and nucleosome spacing activity of the nucleosome remodelling atpase iswi. EMBO J.

[6] Schnitzler GR (2008). Control of nucleosome positions by dna sequence and remodeling machines. Cell Biochem Biophys.

[7] Shahbazian MD, Grunstein M (2007). Functions of site-specific histone acetylation and deacetylation. Annu Rev Biochem.

[8] Nucleosome positioning In: Ranganathan S, Gribskov M, Nakai K, Schönbach C, editors. Encyclopedia of Bioinformatics and Computational Biology. Oxford: Academic Press: 2019. p. 308–17.

[9] Lu Q, Wallrath LL, Elgin SC (1994). Nucleosome positioning and gene regulation. J Cell Biochem.

[10] Svaren J, Horz W (1997). Transcription factors vs. nucleosomes: Regulation of the pho5 promoter in yeast. Trends Biochem Sci.

[11] Liu M-J, Seddon AE, Tsai ZT-Y, Major IT, Floer M, Howe GA, Shiu S-H (2015). Determinants of nucleosome positioning and their influence on plant gene expression. Genome Res.

[12] Pulivarthy SR, Lion M, Kuzu G, Matthews AG, Borowsky ML, Morris J, Kingston RE, Dennis JH, Tolstorukov MY, Oettinger MA (2016). Regulated large-scale nucleosome density patterns and precise nucleosome positioning correlate with v (d) j recombination. Proc Natl Acad Sci.

[13] Satchwell SC, Drew HR, Travers AA (1986). Sequence periodicities in chicken nucleosome core dna. J Mol Biol.

[14] Drew HR, Travers AA (1985). Dna bending and its relation to nucleosome positioning. J Mol Biol.

[15] Lowman H, Bina M. Correlation between dinucleotide periodicities and nucleosome positioning on mouse satellite dna. Biopolymers. 1990; 30(9–10):861–76. https://doi.org/10.1002/bip.360300902. http://arxiv.org/abs/https://onlinelibrary.wiley.com/doi/pdf/10.1002/bip.360300902. 10.1002/bip.360300902 2092816

[16] Giancarlo R, Rombo SE, Utro F (2018). In vitro versus in vivo compositional landscapes of histone sequence preferences in eucaryotic genomes. Bioinformatics.

[17] Kaplan N, K Moore I, Mittendorf Y, J Gossett A, Tillo D, Field Y, M LeProust E, R Hughes T, Lieb J, Widom J, Segal E (2009). The dna-encoded nucleosome organization of a eukaryotic genome. Nature.

[18] Lo Bosco G, Angelini C, Rancoita PM, Rovetta S (2016). Alignment free dissimilarities for nucleosome classification. Computational Intelligence Methods for Bioinformatics and Biostatistics.

[19] Utro F, Di Benedetto V, Corona DFV, Giancarlo R (2015). The intrinsic combinatorial organization and information theoretic content of a sequence are correlated to the DNA encoded nucleosome organization of eukaryotic genomes. Bioinformatics.

[20] Giancarlo R, Rombo SE, Utro F (2019). Dna combinatorial messages and epigenomics: The case of chromatin organization and nucleosome occupancy in eukaryotic genomes. Theor Comput Sci.

[21] Chereji RV, Clark DJ (2018). Major determinants of nucleosome positioning. Biophys J.

[22] Zhao Y, Wang J, Liang F, Liu Y, Wang Q, Zhang H, Jiang M, Zhang Z, Zhao W, Bao Y, Zhang Z, Wu J, Asmann YW, Li R, Xiao J (2018). NucMap: a database of genome-wide nucleosome positioning map across species. Nucleic Acids Res.

[23] Guo S-H, Deng E-Z, Xu L-Q, Ding H, Lin H, Chen W, Chou K-C (2014). inuc-pseknc: a sequence-based predictor for predicting nucleosome positioning in genomes with pseudo k-tuple nucleotide composition. Bioinformatics.

[24] Tahir M, Hayat M (2016). inuc-stnc: a sequence-based predictor for identification of nucleosome positioning in genomes by extending the concept of saac and chou’s pseaac. Mol BioSyst.

[25] Zhang J, Peng W, Wang L (2018). Lenup: learning nucleosome positioning from dna sequences with improved convolutional neural networks. Bioinformatics.

[26] Di Gangi M, Lo Bosco G, Rizzo R (2018). Deep learning architectures for prediction of nucleosome positioning from sequences data. BMC Bioinformatics.

[27] Lo Bosco G, Rizzo R, Fiannaca A, La Rosa M, Urso A. A deep learning model for epigenomic studies. In: 12th International Conference on Signal-Image Technology Internet-Based Systems (SITIS). IEEE: 2016. p. 688–92. 10.1109/sitis.2016.115.

[28] Di Gangi MA, Gaglio S, La Bua C, Lo Bosco G, Rizzo R, Rojas I, Ortuño F (2017). A deep learning network for exploiting positional information in nucleosome related sequences. Bioinformatics and Biomedical Engineering.

[29] Fiannaca A, La Paglia L, La Rosa M, Renda G, Rizzo R, Gaglio S, Urso A (2018). Deep learning models for bacteria taxonomic classification of metagenomic data. BMC Bioinformatics.

[30] Amato D, Di Gangi MA, Lo Bosco G, Rizzo R. Recurrent deep neural networks for nucleosome classification In: Raposo M, Ribeiro P, Sério S, Staiano A, Ciaramella A, editors. Computational Intelligence Methods for Bioinformatics and Biostatistics. Cham: Springer: 2020. p. 118–27.

[31] Di Gesù V, Lo Bosco G, Pinello L, Yuan G-C, Corona DFV (2009). A multi-layer method to study genome-scale positions of nucleosomes. Genomics.

[32] Pinello L, Lo Bosco G, Yuan G-C (2014). Applications of alignment-free methods in epigenomics. Brief Bioinformatics.

[33] Szegedy C, Liu W, Jia Y, Sermanet P, Reed S, Anguelov D, Erhan D, Vanhoucke V, Rabinovich A. Going deeper with convolutions. In: Proceedings of the IEEE Conference on Computer Vision and Pattern Recognition: 2015. p. 1–9. 10.1109/cvpr.2015.7298594.

[34] Chen W, Feng P, Ding H, Lin H, Chou K-C (2016). Using deformation energy to analyze nucleosome positioning in genomes. Genomics.

[35] Liu H, Zhang R, Xiong W, Guan J, Zhuang Z, Zhou S. A comparative evaluation on prediction methods of nucleosome positioning. Brief Bioinforma. 2013; 15. 10.1093/bib/bbt062. 10.1093/bib/bbt062 24023366

[36] Tensorflow. https://www.tensorflow.org/install. Accessed 07 April 2020.

[37] Kingma DP, Ba J. Adam: A method for stochastic optimization. In: 3rd International Conference on Learning Representations, ICLR 2015, San Diego, CA, USA, May 7-9, 2015, Conference Track Proceedings: 2015. http://arxiv.org/abs/1412.6980.

